# Comparative speed of kill of sarolaner (Simparica^™^) and spinosad plus milbemycin oxime (Trifexis^®^) against induced infestations of *Ctenocephalides felis* on dogs

**DOI:** 10.1186/s13071-016-1374-z

**Published:** 2016-02-19

**Authors:** Robert H. Six, William R. Everett, Melanie R. Myers, Sean P. Mahabir

**Affiliations:** Zoetis, Veterinary Medicine Research and Development, 333 Portage St., Kalamazoo, MI 49007 USA; BerTek, Inc, PO Box 606, Greenbrier, AR 72058 USA

**Keywords:** *Ctenocephalides felis*, Sarolaner, Simparica^™^, Spinosad, Milbemycin oxime, Trifexis^®^, Speed of kill, Flea, Dog, Oral, Isoxazoline

## Abstract

**Background:**

Fleas are a ubiquitous ectoparasite infesting dogs and cause direct discomfort, allergic reactions and are responsible for the transmission of several pathogens. The rapid speed of kill of a parasiticide is important to alleviate the direct deleterious effects of fleas, reduce the impact of allergic responses, and break the flea life cycle. In this study, the speed of kill of a novel orally administered isoxazoline parasiticide, sarolaner (Simparica^™^) against fleas on dogs was evaluated and compared with spinosad in combination with milbemycin oxime (Trifexis^®^) for 5 weeks after a single oral dose.

**Methods:**

Twenty-four dogs were randomly allocated to treatment with a single oral dose per product label of sarolaner (2 to 4 mg/kg), spinosad/milbemycin oxime (30 to 60 mg/kg / 0.2 to 0.4 mg/kg), or placebo based on pretreatment flea counts. Dogs were combed and live fleas counted at 8, 12, and 24 h after treatment and subsequent re-infestations on Days 7, 14, 21, 28, and 35. Efficacy (reduction in live flea counts) of each treatment was determined at each time point relative to counts for placebo dogs.

**Results:**

There were no adverse reactions to treatment. A single oral dose of sarolaner provided ≥94.0 % efficacy (based on geometric means) within 8 h of treatment or subsequent weekly re-infestations of fleas to Day 35. By 12 h, fleas were eradicated from all dogs and they remained flea free at 24 h. Significantly greater numbers of live fleas were recovered from spinosad/milbemycin oxime-treated dogs at 8 h from Day 21 to Day 35 (*P* ≤ 0.0085), and at 12 and 24 h on Day 35 (*P* ≤ 0.0002).

**Conclusions:**

In this controlled laboratory evaluation**,** dogs treated with sarolaner had significantly fewer live fleas than spinosad/milbemycin oxime- treated dogs at 8 h after re-infestation from Day 21 after a single oral dose. The rapid and consistent kill of fleas after a single oral dose of sarolaner over 35 days indicates that this treatment should provide highly effective control of flea infestations, relief for dogs afflicted with flea allergy dermatitis, and also reduce the risk of transmission of flea-borne pathogens.

## Background

The cat flea, *Ctenocephalides felis felis,* is the most common ectoparasite of dogs and cats worldwide [1], flea infestation of pets and the home is common and control can be expensive and time consuming [2]. Flea bites can elicit an allergic response, flea allergic dermatitis (FAD), causing intense pruritus and severe skin inflammation [3]. Fleas can also transmit a number of bacterial pathogens and are the intermediate host for filarid and cestode parasites [4–9]. Fleas feed almost immediately on attaining a host [10], and direct irritation and allergic reactions are dependent upon the frequency and duration of feeding [11]. Thus, the rapid kill of fleas is desirable to alleviate both the immediate irritation caused by fleas as well as to reduce associated allergenic responses and the risk of flea-borne pathogen transmission; this may include on-host flea treatment as well as environmental treatment to reduce the household infestation [2]. The speed of kill of adult fleas on the host is also an important factor in the control of infestations, as female fleas do not begin producing eggs until 24 to 48 h after they start feeding [12]. Killing fleas before they lay eggs will, over time, effectively control the environmental infestation.

The use of highly effective parasiticides has allowed the primary means of flea control to be via direct treatment of the pet. Their use, as host-targeted therapies, markedly reduces the severity and prevalence of FAD and has reduced the need to treat indoor and outdoor environments [1]. Orally administered compounds have been introduced that provide rapid systemic control of fleas for up to 24 to 48 h like nitenpyram [13] or for up to a month, spinosad [14]. These products have been widely accepted by veterinarians and dog owners for their efficacy, ease of use and, since these agents are not topically applied, the potential lower exposure of the owner or children to residues. Newer isoxazoline compounds (e.g. fluralaner and afoxolaner) have demonstrated efficacy against fleas and also ticks for a month or longer following a single orally administered dose [15, 16]. Sarolaner (Simparica^™^, Zoetis) is a new isoxazoline effective against fleas and ticks for at least one month following a single oral dose. A laboratory study was conducted to compare the speed of kill of a single dose of sarolaner (Simparica^™^, Zoetis) and spinosad/milbemycin oxime (Trifexis^®^, Elanco) against an existing flea (*C. felis*) infestation and subsequent re-infestations for a period of 5 weeks after treatment.

## Methods

### Ethical approval

This laboratory efficacy study was a masked, negative controlled, randomized complete block design conducted in Arkansas, USA. Procedures were in accordance with the World Association for the Advancement of Veterinary Parasitology (WAAVP) guidelines for evaluating the efficacy of parasiticides for the treatment, prevention and control of flea and tick infestation on dogs and cats [17] and complied with the principles of Good Clinical Practices [18]. The protocol was reviewed and approved by the local Institutional Animal Care and Use Committee. Masking of the study was assured through the separation of functions. All personnel conducting observations or animal care or performing infestations and counts were masked to treatment allocation.

### Animals

Twenty-four, male and female, purpose-bred mongrel dogs from 7 to 35 months of age and weighing from 5.4 to 14.6 kg were used in the study. All dogs had undergone an adequate wash-out period to ensure that no residual ectoparasiticide efficacy remained from any previously administered compound. Each dog was individually identified by electronic transponder or ear tattoo. The dogs were acclimatized to study conditions for a minimum of 14 days before treatment on Day 0. Dogs were individually housed in indoor runs such that no physical contact was possible between them. Dogs were fed an appropriate maintenance ration of a commercial dry canine feed, and water was available *ad libitum*. All dogs were given a physical examination to evaluate general health and suitability for inclusion into the study. General health observations were performed at least once daily from the start of the acclimation period to the end of the study.

### Design

The study followed a randomized complete block design. Dogs were ranked according to decreasing flea counts into blocks of three and within each block a dog was randomly allocated to treatment with placebo, sarolaner, or spinosad/milbemycin oxime. There were eight dogs per treatment group. Dogs were infested with fleas prior to treatment and then weekly following treatment for 5 weeks. Flea counts were conducted at 8, 12, and 24 h after treatment and after each subsequent weekly re-infestation.

### Treatment

Day -2 bodyweights were used to determine the appropriate dose to be administered. On Day 0, dogs received either a placebo tablet, a sarolaner chewable tablet (Simparica^™^) to deliver sarolaner at the minimum label dose of 2 mg/kg (range 2 to 4 mg/kg), or Trifexis^®^ per US label directions (spinosad at 30 to 60 mg/kg plus milbemycin oxime at 0.2 to 0.4 mg/kg). All doses were administered by hand pilling to ensure accurate and complete dosing. Each dog was observed for several minutes after dosing for evidence that the dose was swallowed, and for general health at 1, 4, and 24 h after treatment administration. In order to comply with Trifexis^®^ label requirements, all dogs were offered their regular ration within 30 min of treatment.

### Flea infestation and assessment

The *C. felis* used in the study were from a locally maintained laboratory colony initiated in 2004 with fleas from a laboratory colony in North Carolina, USA. Wild caught fleas locally obtained in Arkansas were introduced into the colony approximately six months prior to study initiation. Flea infestations were performed on Days -7 (host suitability and allocation), -2, 7, 14, 21, 28 and 35. At each infestation a pre-counted aliquot of 100 (±5) adult, unfed *C. felis* were directly applied to the animal which was gently restrained for a few minutes to allow the fleas to penetrate and disperse into the hair coat. Each dog was examined and combed to remove and count fleas at 24 h after the initial host suitability infestation, and at 8, 12, and 24 (±1) hours after treatment and each subsequent weekly re-infestation. Fleas were replaced on the dogs immediately after each 8 and 12 h evaluation, and discarded after the 24 h counts.

Flea counts were performed by personnel trained in the standard procedures in use at the test facility. Commercial fine-toothed flea combs were used. Dogs were combed using repeated strokes initially while standing starting from the head, then proceeding caudally along the dorsum. The dog was then placed on each side and then on its back for combing of the sides and ventral surfaces. After a few combing strokes were completed, the comb was examined and hair and fleas were removed from the comb and all live fleas were counted. Each animal was combed for a minimum of 10 min; if any fleas were recovered in the last minute, combing was continued in one-minute increments until no fleas were detected.

### Statistical analysis

The individual dog was the experimental unit and the primary endpoint was the live flea count. Data for post-treatment flea counts were summarized with arithmetic (AM) and geometric (GM) means by treatment group and time point. Flea counts were transformed (log _e_(count + 1)) prior to analysis to stabilize the variance and normalize the data. Using the PROC MIXED procedure (SAS 9.2, Cary NC), transformed counts were analyzed using a mixed linear model. The fixed effects were treatment, time point and the interaction between time point and treatment by time point. The random effects included block, block by treatment interaction, and error. Testing was two-sided at the significance level α = 0.05.

The assessment of efficacy was based on the percent reduction in the arithmetic and geometric mean live flea counts relative to placebo calculated using Abbott’s formula:

$$ \%\ \mathrm{reduction}=100 \times \frac{\mathrm{mean}\ \mathrm{count}\ \left(\mathrm{placebo}\right)\hbox{--} \mathrm{mean}\ \mathrm{count}\ \left(\mathrm{treated}\right)}{\mathrm{mean}\ \mathrm{count}\ \left(\mathrm{placebo}\right)} $$

## Results

There were no treatment-related adverse events during the study. Placebo-treated dogs maintained good flea infestations throughout the study and these counts were maintained even following the combing and re-infestation procedures at 8 and 12 h (Tables 1, 2 and 3).

**Table 1:** Mean live flea counts and efficacy relative to placebo at 8 h after treatment and post-treatment re-infestations for dogs treated with a single oral dose of sarolaner or spinosad/milbemycin oxime on Day 0

Treatment		Day of treatment or re-infestation					
		0	7	14	21	28	35
Placebo	Range	80–100	45–100	71–100	70–100	85–100	74–100
	A. mean	91.8	86.0	87.4	87.8	96.1	86.6
	G. mean^1^	91.5^a^	83.9^a^	86.7^a^	87.3^a^	96.0^a^	86.3^a^
Sarolaner	Range	0–0	0–1	0–0	0–2	0–18	0–56
	A. mean	0.0	0.1	0.0	0.3	3.0	14.9
	Efficacy (%)	100	99.9	100	99.7	96.9	82.8
	G. mean^1^	0.0^b^	0.1^b^	0.0^b^	0.1^c^	0.8^c^	5.2^c^
	Efficacy (%)	100	99.9	100	99.8	99.1	94.0
	*P*-value vs.placebo	<0.0001	<0.0001	<0.0001	<0.0001	<0.0001	<0.0001
Spinosad/milbemycin oxime	Range	0–0	0–0	0–0	0–50	0–69	0–73
	A. mean	0.0	0.0	0.0	9.1	22.3	38.4
	Efficacy (%)	100	100	100	89.6	76.9	55.7
	G. mean^1^	0.0^b^	0.0^b^	0.0^b^	2.6^b^	5.3^b^	23.1^b^
	Efficacy (%)	100	100	100	97.0	94.4	73.2
	*P*-value vs. placebo	<0.0001	<0.0001	<0.0001	<0.0001	<0.0001	0.0021
	*P*-value vs. sarolaner	1.000	0.8420	1.000	0.0085	0.0049	0.0020

^1^Geometric means within columns with the same superscript are not significantly different (*P* > 0.05)

**Table 2:** Mean live flea counts and efficacy relative to placebo at 12 h after treatment and post-treatment re-infestations for dogs treated with a single oral dose of sarolaner or spinosad/milbemycin oxime on Day 0

Treatment		Day of treatment or re-infestation					
		0	7	14	21	28	35
Placebo	Range	84–100	19–96	80–100	67–100	80–97	66–98
	A. mean	92.0	77.0	87.3	86.6	87.3	81.8
	G. mean^1^	91.8^a^	70.7^a^	87.1^a^	85.9^a^	87.1^a^	81.2^a^
Sarolaner	Range	0–0	0–0	0–0	0–0	0–0	0–0
	A. mean	0.0	0.0	0.0	0.0	0.0	0.0
	Efficacy (%)	100	100	100	100	100	100
	G. mean^1^	0.0^b^	0.0^b^	0.0^b^	0.0^b^	0.0^b^	0.0^c^
	Efficacy (%)	100	100	100	100	100	100
	*P*-value vs. placebo	<0.0001	<0.0001	<0.0001	<0.0001	<0.0001	<0.0001
Spinosad/milbemycin oxime	Range	0–0	0–0	0–0	0–36	0–64	0–68
	A. mean	0.0	0.0	0.0	4.5	9.5	27.9
	Efficacy (%)	100	100	100	94.8	89.1	65.9
	G. mean^1^	0.0^b^	0.0^b^	0.0^b^	0.6^b^	1.3^b^	8.2^b^
	Efficacy (%)	100	100	100	99.3	98.5	90.0
	*P*-value vs. placebo	<0.0001	<0.0001	<0.0001	<0.0001	<0.0001	<0.0001
	*P*-value vs. sarolaner	1.000	1.000	1.000	0.2996	0.0538	<0.0001

^1^Geometric means within columns with the same superscript are not significantly different (*P* > 0.05)

**Table 3:** Mean live flea counts and efficacy relative to placebo at 24 h after treatment and post-treatment re-infestations for dogs treated with a single oral dose of sarolaner or spinosad/milbemycin oxime on Day 0

Treatment		Day of treatment or re-infestation					
		0	7	14	21	28	35
Placebo	Range	69–100	11–96	73–89	60–100	69–94	15–100
	A. mean	83.1	75.5	80.5	85.8	84.0	78.5
	G. mean^1^	82.7^a^	65.8^a^	80.3^a^	84.7^a^	83.7^a^	70.1^a^
Sarolaner	Range	0–0	0–0	0–0	0–0	0–0	0–0
	A. mean	0.0	0.0	0.0	0.0	0.0	0.0
	Efficacy (%)	100	100	100	100	100	100
	G. mean^1^	0.0^b^	0.0^b^	0.0^b^	0.0^b^	0.0^b^	0.0^b^
	Efficacy (%)	100	100	100	100	100	100
	*P*-value vs. placebo	<0.0001	<0.0001	<0.0001	<0.0001	<0.0001	<0.0001
Spinosad/milbemycin oxime	Range	0–0	0–0	0–0	0–6	0–54	0–68
	A. mean	0.0	0.0	0.0	0.8	6.8	19.6
	Efficacy (%)	100	100	100	99.1	92.0	75.0
	G. mean^1^	0.0^b^	0.0^b^	0.0^b^	0.3^b^	0.7^b^	4.3^c^
	Efficacy (%)	100	100	100	99.7	99.2	93.9
	*P*-value vs. placebo	<0.0001	<0.0001	<0.0001	<0.0001	<0.0001	<0.0001
	*P*-value vs. sarolaner	1.000	1.000	1.000	0.5758	0.2499	0.0002

^1^Geometric means within columns with the same superscript are not significantly different (*P* > 0.05)

At the 8-h time point, both treatments resulted in significantly lower flea counts than placebo-treated dogs (*P* ≤ 0.0021) throughout the study (Table 1). Treatment with sarolaner resulted in significantly lower flea counts than spinosad/milbemycin oxime at 8 h on Days 21, 28, and 35 (*P* < 0.0085). The sarolaner treatment provided greater and more consistent efficacy at 8 h, with efficacy >96.9 % (GM and AM) from treatment through Day 28. Efficacy for spinosad/milbemycin oxime was <95 % on Days 21 to 35 (AM) and on Days 28 and 35 (GM) (Table 1).

**Fig. 1:**
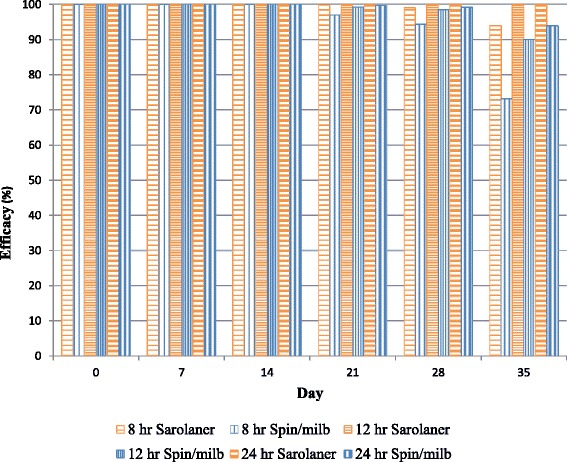
Percent efficacy based on geometric mean counts relative to placebo at 8,12 and 24 h after treatment and weekly post-treatment re-infestations of fleas for dogs treated with a single oral dose of sarolaner or spinosad/milbemycin oxime on Day 0

At the 12-h time point, both treatments resulted in significantly lower flea counts than placebo-treated dogs (*P* ≤ 0.0001) throughout the study (Table 2). Flea counts were significantly lower for sarolaner-treated dogs than spinosad/milbemycin oxime-treated dogs on Day 35 (*P* < 0.0001). Efficacy was very high for sarolaner with all dogs being flea-free on all study days, while live fleas were detected on spinosad/milbemycin oxime treated dogs from Day 21 through Day 35. Efficacy of spinosad/milbemycin oxime declined as the study progressed (Fig. 1); at Days 21 to 35 efficacy for spinosad/ milbemycin oxime-treated dogs was <95 % (AM), and on Day 35 (GM) (Table 2).

At the 24-h time point, both treatments resulted in significantly lower flea counts than placebo-treated dogs (*P* ≤ 0.0001) throughout the study (Table 3). Flea counts were significantly lower for sarolaner-treated dogs than spinosad/milbemycin oxime-treated dogs on Day 35 (*P* = 0.0002). Sarolaner remained 100 % effective at 24 h post treatment, with all dogs flea free from treatment through Day 35, while live fleas were detected on spinosad/milbemycin oxime-treated dogs from Day 21 onwards. Efficacy for spinosad/milbemycin oxime declined below 95 % by Day 28 based on AM and on Day 35 based on GM (Table 3, Fig. 1).

## Discussion

A single oral dose of sarolaner provided rapid reduction of an existing infestation of fleas as well as subsequent weekly re-infestations for 35 days; efficacy was ≥99.1 % based on GM (≥96.9 %, based on AM) from treatment through Day 28 and 94.0 % (GM) or 82.8 % (AM) on Day 35 at 8 h after treatment or re-infestation. By 12 and 24 h after treatment or re-infestation, efficacy of sarolaner was 100 % (all dogs were free of fleas) for the entire 35 day period. A similar speed of kill was attained for only 14 days at 8 h after treatment or re-infestation with a single oral dose of spinosad/milbemycin oxime, and from Day 21 onwards its speed of kill was significantly slower than that of sarolaner. Even at 12 and 24 h after re-infestation, significantly more live fleas were found on spinosad/milbemycin oxime-treated dogs on Day 35 and efficacy at 12 h declined below 95 % from Day 21 onwards (AM) and on Day 35 (GM), and at 24 h efficacy was <95 % from Day 28 onwards (AM) and on Day 35 (GM). In contrast sarolaner-treated dogs were free of fleas (100 % AM and GM) at 12 and 24 h for the entire 35 days.

A rapid onset of activity and consistent speed of kill for any parasiticide providing flea control is essential to ensure that any newly acquired fleas are rapidly eliminated before they can reproduce to help eliminate the environmental infestation, decrease the likelihood of transmission of vector-borne disease and assist in the management of flea allergic dermatitis. This provides the pet with rapid relief from the irritation and debilitating effects of the existing infestation and protects it from new infestations. A single oral treatment of sarolaner at the proposed commercial dose of 2 to 4 mg/kg resulted in the rapid reduction of an existing flea infestation as well as rapid kill of newly infested fleas for at least 35 days, and efficacy was more consistent over the full month with significantly faster kill of fleas than spinosad/milbemycin oxime from Day 21 onwards.

## Conclusions

Both products resulted in rapid control of an existing flea infestation. Fleas were eliminated from all dogs within 8 h and dogs remained flea-free at 24 h. Against re-infestations, efficacy of sarolaner at 8 h was significantly superior to spinosad/milbemycin oxime on Days 21 to 35 and at 12 and 24 h on Day 35. The efficacy of spinosad/ milbemycin oxime waned at the end of the month long treatment interval, while sarolaner maintained high efficacy with all dogs being flea free by 12 h from treatment through Day 35. The rapid and consistent speed of kill of fleas over a period of 35 days makes sarolaner chewable tablets (Simparica^™^) an excellent option for monthly flea control that will reduce the direct irritation caused by flea infestation, assist in the prevention of FAD, and reduce the risk of flea-borne diseases.
